# The possibility of clinical bonding between metal/ceramic brackets to zirconia: *in vitro* study

**DOI:** 10.3389/fbioe.2024.1354241

**Published:** 2024-01-15

**Authors:** Yichun Hu, Jiayang Gao, Xinyue Huang, Yutong Li, Ziyi Chen, Desong Zhan, Hidehiko Sano, Ricardo M. Carvalho, Jiale Fu

**Affiliations:** ^1^ School and Hospital of Stomatology, China Medical University, Shenyang, China; ^2^ Department of Dental Materials Science, The Second Department of Prosthodontics, School and Hospital of Stomatology, China Medical University, Shenyang, China; ^3^ Department of Restorative Dentistry, Division of Oral Health Science, Faculty of Dental Medicine, Hokkaido University, Sapporo, Japan; ^4^ Department of Oral Biological and Medical Sciences, Division of Biomaterials, Faculty of Dentistry, University of British Columbia, Vancouver, BC, Canada

**Keywords:** shear bond strength (SBS), ceramic bracket, zirconia, resin cement, metal bracket, storage condition

## Abstract

**Objective:** The present study aimed to assess the bond strength and durability of six bonding agents concerning their application to metal or ceramic brackets and zirconia.

**Materials and Methods:** Six resin cement bonding agents (XT, XTS, RSBU, RGBU, SBPM, and GMP) were chosen for this investigation. Specimens were either stored in distilled water at 37°C for 24 h or subjected to 5,000 thermocycles before conducting a Shear Bond Strength (SBS) test. Statistical analysis of the SBS data was performed using three-way ANOVA and Games-Howell tests (α = 0.05). The Adhesive Remnant Index was examined, and the debonding surface details on brackets and zirconia were observed.

**Results:** For metal brackets, all groups demonstrated clinically acceptable bond strength, irrespective of storage conditions, except for the XT group. Regarding ceramic brackets, all groups displayed acceptable bond strength after 24 h of water storage. However, following thermocycling, a significant decrease in SBS was noted across all groups (*p* < 0.05), with SBPM exhibiting a higher bond strength. Three-way ANOVA analysis indicated that SBS values were notably influenced by each factor, and an interaction among the three independent variables was observed (*p* = 0.000).

**Conclusion:** The reliable bond strength between ceramic brackets and zirconia was significantly lower after thermocycling compared to that of metal brackets and zirconia. SBPM exhibited consistent and robust bond strength between ceramic/metal brackets and zirconia across various storage conditions. Furthermore, the HEMA-free adhesive demonstrated a potentially more consistent bonding performance compared to the HEMA-containing adhesive employed in this study.

## 1 Introduction

Zirconia (ZrO2) has gained widespread use in dentistry for fixed dental prostheses (FDPs), single crowns, bridge restorations, and implant abutments (Ju et al., 2020). Its exceptional mechanical strength sets it apart from other conventional ceramic materials (Zhang and Lawn, 2018). Additionally, Zirconia exhibits favorable biocompatibility, aesthetic properties, and high resistance to corrosion (Gautam et al., 2016; Zarone et al., 2019). The popularity of zirconia restorations has surged due to their convenient milling from prefabricated disks using CAD/CAM devices (Ju et al., 2019; Goracci et al., 2022).

Zirconia crystals exist in various patterns: monoclinic (M), cubic (C), and tetragonal (T) structures (Nistor et al., 2019). At ambient temperature, the monoclinic phase is most stable but transforms into tetragonal and cubic phases upon heating (Hanawa, 2020). Yttrium-stabilized zirconia (YSZ), also known as tetragonal zirconia polycrystal (TZP), achieves enhanced molecular stability by combining ZrO2 with Y2O3(Nistor et al., 2019; Hanawa, 2020). The typical yttria content in dental YSZ ranges between 3 and 5 mol% (Vult von Steyern et al., 2022). Increasing the yttria content enhances zirconia’s translucency for anterior teeth restoration, but it compromises strength and toughness (Vult von Steyern et al., 2022). In clinical practice, zirconia is predominantly used for porcelain-fused-to-zirconia crowns in anterior teeth, while full zirconia crowns are favored for posterior teeth (Makhija et al., 2016; Rauch et al., 2021). Despite zirconia’s microstructural characteristics, chemical inertness, and biocompatibility, establishing reliable bonding between zirconia and resin cement remains challenging (Lima et al., 2019). Silica-based porcelains are preferred due to superior translucency and their ability to be acid-etched and silanized, enhancing adhesion and reinforcing resin bonding (Zhang and Lawn, 2018).

The rising demand for dental aesthetics has led to an increased number of individuals seeking orthodontic treatment, including adults with a history of fixed prosthetic treatments (Babaee Hemmati et al., 2022). Orthodontic brackets are categorized as metal or ceramic brackets. Ceramic brackets were developed to meet the demand for improved aesthetics (Urichianu et al., 2022). Despite their aesthetic appeal, ceramic brackets exhibit lower bond strength to enamel, acrylic, and porcelain surfaces compared to traditional metal brackets (Pinho et al., 2020). Moreover, ceramic brackets are prone to fracture and may cause irreversible tooth damage during debonding (Alexopoulou et al., 2020). The demographic of adult patients seeking orthodontic treatment is on the rise (Jawad et al., 2015; Hellak et al., 2016).

As ceramic restorations, including zirconia restorations, are frequently performed in adults, the bonding strategy for different brackets and restoration surfaces becomes a pertinent concern (Pinho et al., 2020). Numerous chair-side challenges can arise when orthodontic brackets are bonded to zirconia ceramic surfaces. These challenges include insufficient familiarity with bonding techniques, lack of knowledge about resin cement or bonding products, and inconvenient treatment methods. For instance, clinicians may suggest replacing a ceramic crown with a CAD/CAM resin crown during orthodontic treatment to achieve better bonding performance, subsequently remaking the ceramic crown (Blakey and Mah, 2010). Enhancing the bonding performance between zirconia and metal or ceramic brackets to cater to orthodontic treatment needs could significantly benefit patients. However, scarce previous studies have explored the durability of various bonding agents between different brackets and zirconia.

Hence, this study aims to evaluate the bonding performance and durability of six different bonding agents concerning their application to metal or ceramic brackets and zirconia. The null hypotheses were as follows: (1) no significant difference exists in the bond strength of different bonding agents; (2) the storage condition does not affect the bond strength of the six bonding agents; and (3) metal/ceramic brackets achieve equivalent bonding durability on zirconia.

## 2 Materials and methods

### 2.1 Bonding agents and brackets

CAD/CAM-produced zirconia cube specimens (48 in total, Aidite Technology, Qinhuangdao, China) with a length of 2 cm were acquired for the study. These zirconia samples were randomly divided into six experimental groups based on the use of specific bonding agents:

• Group 1 (XT): Transbond™ XT Light Cure adhesive paste (XTL) + Transbond™ XT Light Cure Orthodontic Adhesive Primer (XTP)
• Group 2 (XTS): Transbond™ XT Light Cure adhesive paste (XTL) + Single Bond Universal (SBU)
• Group 3 (RSBU): Rely X™ Ultimate Clicker Adhesive Resin Cement (RUC) + Single Bond Universal (SBU)
• Group 4 (RGBU): Rely X™ Ultimate Clicker Adhesive Resin Cement (RUC) + Gluma Bond Universal (GBU)
• Group 5 (SBPM): Superbond C&B (SB) + Porcelain liner M (PLM)
• Group 6 (GMP): GC G-CEM ONE (GCO) + G-Multi Primer (MP)

Please refer to Table 1 for detailed information on the chemical composition and application procedures of the bonding agents and cleaning paste used in this study.

**TABLE 1:** The chemical composition and application procedure of the bonding agents and cleaning paste used in present study.

Code	Materials (Manufacturer/Lot No)	Chemical formulation	Application procedure
XTP	Transbond™ XT Light Cure Orthodontic Adhesive Primer (3M Unitek, Monrovia, CA, United States/NE66698)	Bis-GMA and TEGDMA, triphenylantimony, CQ, DMAEMA	1. Place 1 drop of primer on the surface of zirconia
			2. Gently blow dry the primer for 15 s to a thin uniform coat
			3. Light curing for 20 s
XTL	Transbond™ XT Light Cure adhesive paste (3M Unitek/NE79372)	Bis-GMA, TEGDMA, Bis-EMA quartz, silicon dioxide, canforquinone, DMAEMA	1. Apply appropriate amount of paste to the bracket base with syringe
			2. Lightly place the bracket onto zirconia surface
			3. Remove excess adhesive and light curing for 20 s on each side of the bracket at a distance of approximately 5 mm from the surface
SBU	Single Bond Universal (3M ESPE, St. Paul, MN, United States/11220A)	10-MDP, dimethacrylate resins, HEMA, polyalkenoic acid copolymer, filler, ethanol, water, initiators, silane	1. Apply the adhesive to the zirconia surface and brackets base
			2. Gently blow dry the adhesive for 15 s to a thin uniform coat
			3. Light cure for 20 s
GBU	Gluma Bond Universal (Kulzer, Hanau, Germany/K010046)	10-MDP, 4-META, Methacrylates, acetone and water	1. Apply the adhesive to the zirconia surface and brackets base
			2. Gently blow dry the adhesive for 15 s to a thin uniform coat
			3. Light cure for 20 s
RUC	Rely X™ Ultimate Clicker Adhesive Resin Cement (3M ESPE/9037935)	Base paste: methacrylate monomers, radiopaque, silanated fillers, initiator components, stabilizers, rheological additives	1. Apply appropriate amount of paste to the bracket base with syringe
		Catalyst paste: methacrylate monomers, radiopaque, alkaline (basic) fillers, initiator components, stabilizers, pigments, rheological additives, fluorescence dye, dual-cure activator for single bond universal adhesive	2. Immediately after applying adhesive, lightly place the bracket onto zirconia surface
			3. Remove excess adhesive and light cure for 20 s on each side of the bracket at a distance of approximately 5 mm from the surface
PLM	Porcelain liner M (Sun Medical Company, Kyoto, Japan/VF1F, VR1)	liquid A: MMA,4-META; liquid B: MMA, MPTS	1. Apply 1 drop of liquid A and liquid B into mixing plate which was cooled in the refrigerator in advance. Gently mixed A and B together
			2. Apply the Liner M mixture to the zirconia surface and ceramic bracket base
			3. Gently blow dry the primer for 15 s to a thin uniform coat
SB	Superbond C&B (Sun Medical Company/EV12)	TBB, MMA, 4-META, red treatment agent (65%phosphoric acid), green treatment agent (10%citric acid, 3%Ferric chloride), PMMA	1. Gently mixed the base material, catalyzer and L-type Radiopaque in the proportion of 4:1:1
			2. Apply appropriate amount of mixture to the bracket base. 3. Place the bracket onto zirconia surface and fix it
			3. Place the bracket onto zirconia surface and fix it
			4. Gently remove the excess cement around the bracket base without disturbing the bracket.
MP	G-Multi PRIMER (GC, Tokyo, Japan/2207081)	MPTMS, 10-MDP, MDTP, BisGMA, TEGDMA, ethanol	1. Apply the primer to the zirconia surface and brackets base respectively
			2. Gently blow dry the adhesive for 15 s to a thin uniform coat
GCO	G-CEM ONE (GC/2202182)	A: Silicate glass powder, 3-Methacryloxypropyltrimethoxysilane, 2-Propenoic-3,3,3-d3 acid, methyl ester, Silicon dioxide	1. Apply appropriate amount of cement to the bracket after hand-mixing
		B: Silicon dioxide, 3-Methacryloxypropyltrimethoxysilane, 2-Propenoic-3,3,3-d3 acid, methyl ester, 12-Methacryloyldodeylphosphate, Cumyl hydroperoxide	2. Lightly place the bracket onto zirconia surface and fix it
			3. Remove excess adhesive and light cure for 20 s on each side of the bracket at a distance of approximately 5 mm from the surface
IVO	Ivoclean (Ivoclar-Vivadent, Schaan, Liechtenstein/Y49501)	Zirconium oxide, water, polyethylene glycol, sodium hydroxide, pigments, additives	1. Apply ivoclean to prepared zirconia surface for 60 s
			2. Rinse with distilled water for 15 s and gently blow dry

HEMA, 2-hydroxyethyl methacrylate; TEGDMA, triethyleneglycol dimethacrylate; Bis-GMA, bisphenol A-diglycidyl methacrylate; Bis-EMA, bisphenol A ethoxylated dimethacrylate; 10-MDP, 10-methacryloyloxydecyl dihydrogen phosphate; CQ, camphorquinone; DMAEMA, dimethylaminoethyl methacrylate; MPTS, (3-Mercaptopropyl) trimethoxysilane; 4-META, 4-methacryloxyethyl trimellitate anhydride; MMA, methyl methacrylate; TBB, tributylborane; PMMA, poly (methyl methacrylate); MDTP, methacryloyloxydecyl dihydrogen thiophosphate; MPTMS, γ-methacryloxypropyl trimethoxysilane.

The zirconia cube specimens in each group were divided into two subgroups, each accommodating two types of brackets: metal brackets (Victory Series, 3M Unitek, United States) and ceramic brackets (Maia Series, Protect, Zhejiang, China). A total of 216 metal brackets and 216 ceramic brackets were utilized. The mean base surface areas of the metal and ceramic brackets were 11.94 mm^2^ and 14.82 mm^2^.

### 2.2 Surface preparation

To prepare the bonding surfaces on the zirconia cube, sandblasting with 125 μm aluminum oxide particles was conducted for 60 s at a distance of 1 cm and 2.8 bar pressure. Subsequently, all specimens underwent ultrasonic cleaning in distilled water for 5 min and gentle air drying for 15 s. The application of Ivoclean (IVO) followed the manufacturer’s instructions.

### 2.3 Application procedure

The same pressure (5 N) was applied to each bracket using a consistent clamp across all groups. The application procedures for light curing (Kerr Demi Plus, Orange, CA, United States) and cement removal were standardized and detailed in Table 1.

Each group comprised a total of 72 bracket specimens, divided into two subgroups (metal and ceramic brackets) with 36 specimens each.

### 2.4 Shear bond strength (SBS) test

**FIGURE 1:**
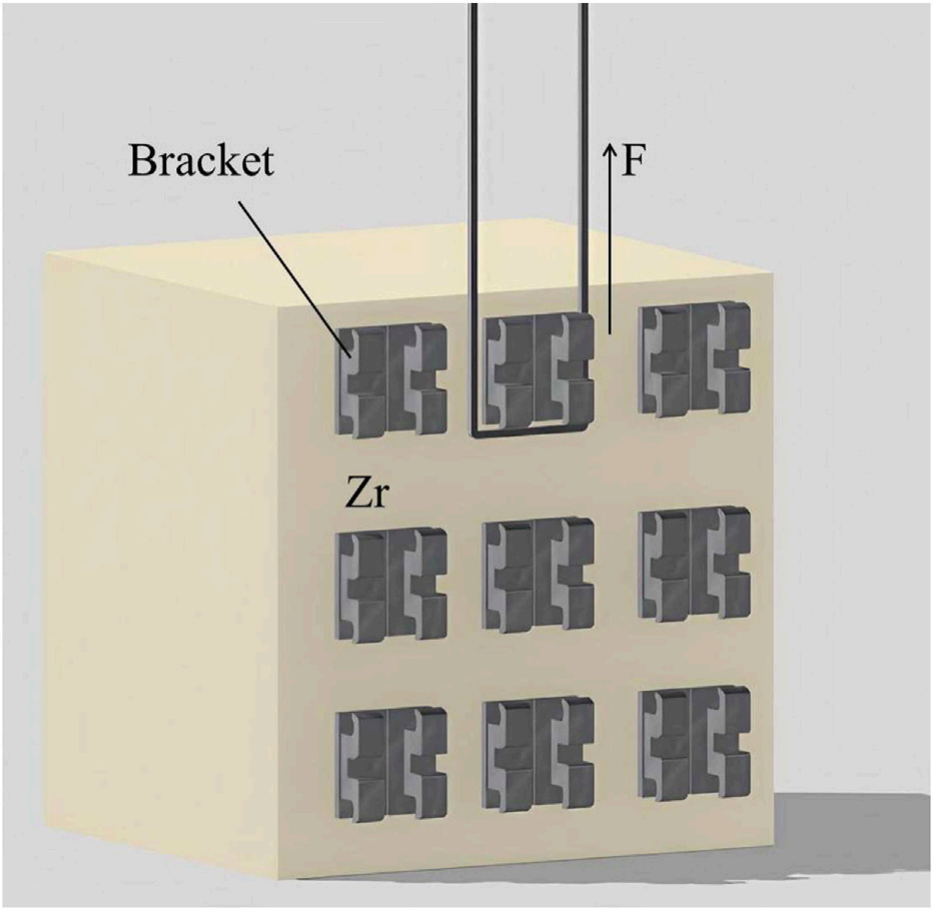
Diagram of specimen setting for SBS Test.

Half of the brackets in each subgroup were tested after storing in distilled water at 37°C for 24 h, while the rest underwent 5,000 cycles of thermocycling between 5°C and 55°C in a thermocycling device (SD Mechatronik, Feldkirchen-Westerham, Germany). The SBS test was conducted using a universal testing machine (WD-200 Weidu, Wenzhou, China) with a crosshead speed of 1 mm/min (Figure 1). The SBS formula used was P (MPa) = F (N)/S (mm^2^). Out of 18 results in each group, the highest 4 and lowest 4 data points were discarded, and the remaining 10 were considered for analysis (n = 10).

### 2.5 Adhesive remnant index (ARI) score

The ARI scoring was performed based on the amount of remaining cement on the zirconia surface with the help of a dental digital camera (EyeSpecial C-IV, shofu, Koyoto, Japan). The score is represented by a scale with 5 levels (Score A to Score E) as follows:

Score A: almost all the cement remained on the zirconia surface;

Score B: more than 90% of the cement remained on the zirconia surface;

Score C: more than 10% but less than 90% of the cement remained on the zirconia surface;

Score D: less than 10% of the cement remained on the zirconia surface;

Score E: no cement remained on the zirconia surface.

**FIGURE 2:**
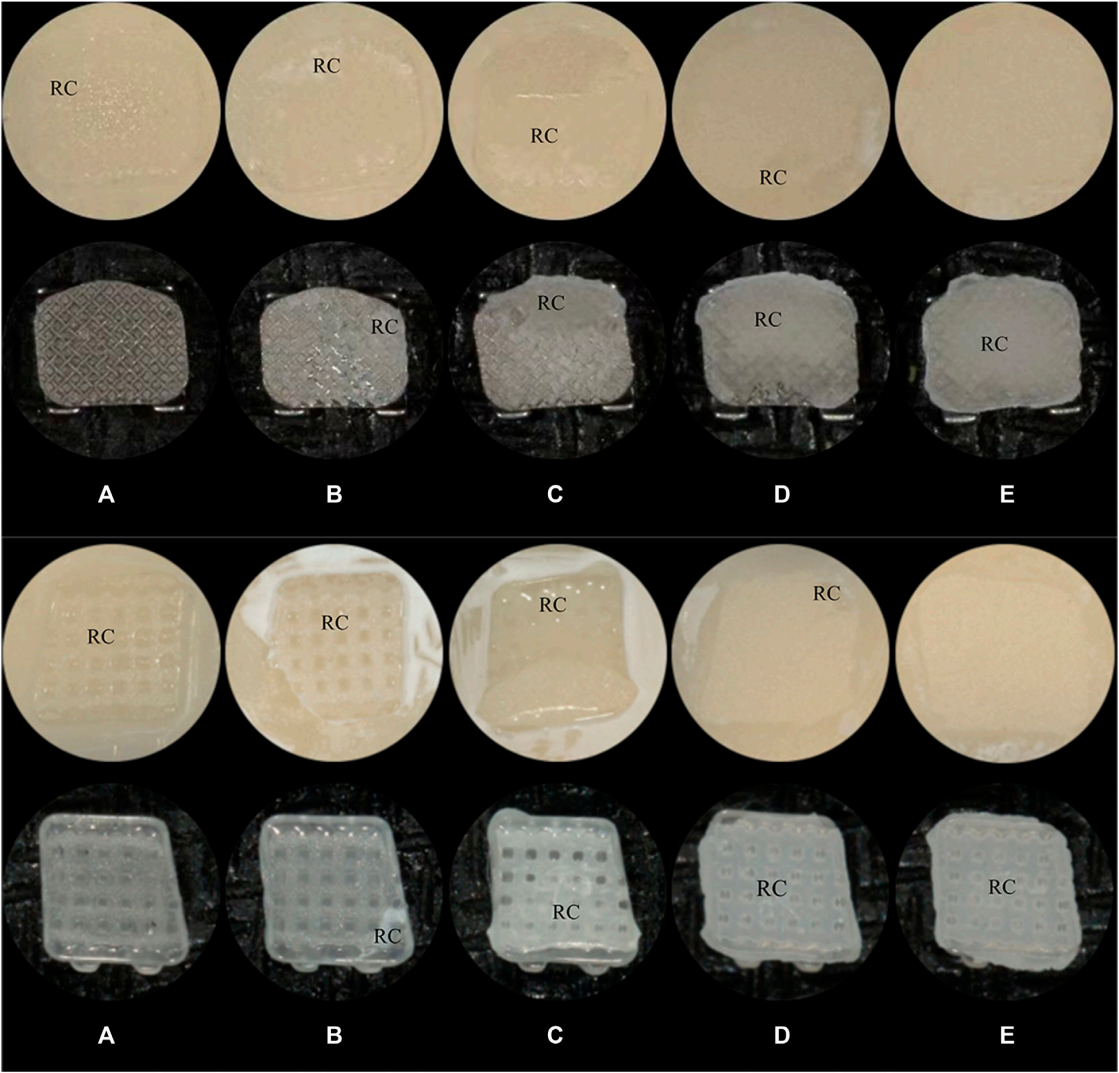
The ARI scores**(A)**: score A; **(B)**: score B; **(C)**: score C; **(D)**: score D; **(E)**: score E; RC: Resin Cement. **(A)** Metal Brackets **(B)** Ceramic Brackets.

Images were scored by three calibrated examiners, and a majority opinion was adopted in cases of disagreement. Figure 2 demonstrates the ARI score on the samples.

### 2.6 Statistical analysis

Statistical analysis involved three-way ANOVA (bonding agents, storage conditions, and brackets) and the Games-Howell test using SPSS version 26.0, with a significance level of α = 0.05.

### 2.7 Surface evaluation

The debonding bracket base surface was analyzed using field emission scanning electron microscopy (FE-SEM) and energy dispersive X-ray spectrometry (EDS; Phenom Pharos G2, Netherlands) to determine elemental composition and distribution.

## 3 Results

### 3.1 Shear bond strength (SBS)

The SBS mean values and standard deviations for each group are summarized in Table 2. Notably, RSBU, SBPM, and GMP exhibited higher SBS for metal bracket groups after 24 h of water storage, with no significant difference among these groups (*p* > 0.05). However, RSBU’s SBS decreased significantly after thermocycling, while SBPM and GMP remained unchanged.

**TABLE 2:** SBS values (MPa) for six bonding agents using different brackets in two different storage conditions (mean ± SD).

Bracket	Storage	XT	XTS	RSBU	RGBU	SBPM	GMP
Metal brackets	24H	3.91 ± 0.81^1,A^	6.61 ± 1.49^1,B^	15.62 ± 1.19^1,C^	12.36 ± 1.01^1,D^	16.31 ± 2.67^1,C^	15.62 ± 1.60^1,C^
		*p* < 0.05	*p* < 0.05	*p* < 0.05	*NS*	*NS*	*NS*
	5,000 cycles	0.25 ± 0.44^3,A^	12.64 ± 1.28^3, BC^	12.44 ± 0.59^3,B^	12.71 ± 1.84^3, BC^	14.91 ± 1.96^3, CD^	14.93 ± 0.93^3,D^
Ceramic brackets	24H	6.82 ± 1.17^2,A^	6.64 ± 2.03^1,A^	8.37 ± 1.44^2, AB^	10.69 ± 2.57^1, BC^	14.89 ± 1.54^1,D^	10.81 ± 1.7^2,C^
		*p* < 0.05	*p* < 0.05	*p* < 0.05	*p* < 0.05	*p* < 0.05	*p* < 0.05
	5,000 cycles	0.61 ± 0.12^4,A^	2.33 ± 0.91^4,B^	2.78 ± 0.90^4,B^	2.58 ± 0.87^4,B^	10.65 ± 1.69^4,C^	2.36 ± 0.95^4,B^

*NS* indicates no significance between storage periods for each type of bracket (*p* > 0.05).

Identical capital letters indicate no significant differences among materials for each storage period (*p* > 0.05).

Identical numbers indicate no significant differences between brackets for each storage condition (*p* > 0.05).

For ceramic brackets, SBPM displayed higher SBS after 24 h of water storage but significantly declined post-thermocycling, akin to other bonding agents (*p* < 0.05). Comparatively, ceramic brackets demonstrated a more significant decrease in SBS after thermocycling in contrast to metal brackets. Generally, the SBS of ceramic brackets was lower than that of metal brackets after both 24 h of water storage (*p* > 0.05) and thermocycling (*p* < 0.05), except for XTS group results (*p* > 0.05) post-24 h of water storage and XT group outcomes under both storage conditions (*p* < 0.05).

SBPM showcased higher SBS values after thermocycling, irrespective of bracket type, while XT exhibited the lowest SBS in the metal bracket group. XTS displayed a significant increase in SBS for metal brackets post-thermocycling (*p* < 0.05).

The SPSS analysis (Table 3) revealed significant influences on SBS values by brackets, bonding agents, and storage conditions (*p* < 0.001). Interactions were observed among brackets, bonding agents, and storage conditions (*p* < 0.001).

**TABLE 3:** Three-way ANOVA analysis result.

Source	df	Mean square	F	P
Corrected model	23	288.012	135.007	0.000
Intercept	1	19770.251	9267.415	0.000
Brackets	1	1439.375	674.715	0.000
Bonding agents	5	581.216	272.448	0.000
Storage conditions	1	649.15	304.293	0.000
Bonding agents * Brackets	5	149.857	70.246	0.000
Storage conditions * Brackets	1	491.491	230.389	0.000
Bonding agents * Storage conditions	5	46.589	21.839	0.000
Bonding agents * Storage conditions * Brackets	5	31.189	14.62	0.000

Regarding the SBS results in Tables 4, 5, metal brackets displayed significantly higher SBS than ceramic brackets after thermocycling (*p* < 0.05). Also, RSBU, RGBU, SBPM, and GMP exhibited significantly higher SBS after 24 h of water storage compared to XT and XTS (*p* < 0.05). XT demonstrated notably lower SBS than other groups post-thermocycling (*p* < 0.05). SBPM consistently demonstrated higher SBS irrespective of storage conditions.

**TABLE 4:** The mean SBS values (MPa) of all six bonding agents in the different experimental groups (mean ± SD).

Storage	Metal brackets	Ceramic brackets
24H	11.74 ± 5.08^a^	9.70 ± 3.36^a^
5,000 cycles	11.31 ± 5.25^a^	3.55 ± 3.42^b^

Identical lower case letters indicate no significant differences between values (*p* > 0.05).

**TABLE 5:** The mean shear bond strength values (MPa) of all brackets in the different experimental groups (mean ± SD).

Storage	XT	XTS	RSBU	RGBU	SBPM	GMP
24H	5.36 ± 1.78^A^	6.63 ± 1.73^A^	11.99 ± 3.94^B^	11.53 ± 2.08^B^	15.60 ± 2.24^C^	13.21 ± 2.97^BC^
	*p* < 0.05	*NS*	*p* < 0.05	*p* < 0.05	*p* < 0.05	*p* < 0.05
5000cycles	0.43 ± 0.37^A^	7.48 ± 5.40^B^	7.61 ± 5.01^B^	7.65 ± 5.38^B^	12.78 ± 2.82^C^	8.65 ± 6.51^BC^

*NS* indicates no significance in SBS, between storage periods for each material (*p* > 0.05).

Identical capital letters indicate no significant differences in SBS among materials for each storage period (*p* > 0.05).

### 3.2 Failure modes

**FIGURE 3:**
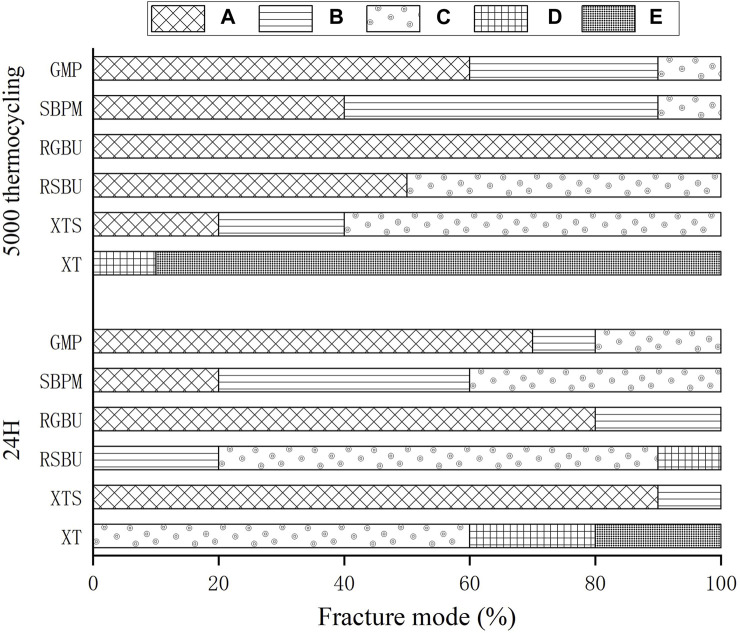
Percentages (%) of the different failure modes after SBS test of metal brackets bonded to zirconia. A to E correspond to Score A to Score E.

**FIGURE 4:**
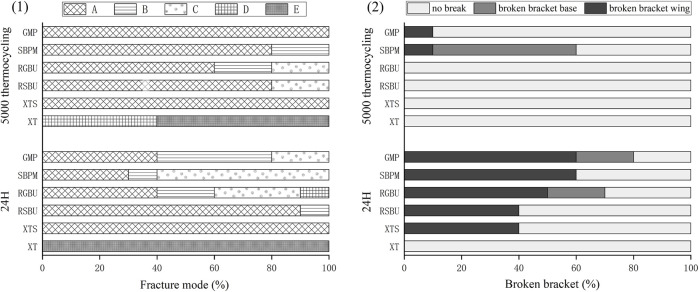
Results of ARI and breakage of ceramic brackets. (1) Percentages (%) of the different failure modes after shear bond strength test of ceramic brackets bonded to zirconia. (2) Percentages (%) of breakage of different ceramic brackets.

The distribution of failure modes for metal and ceramic bracket groups are visualized in Figures 3, 4, respectively. Under 24 h water storage, ARI scores for various groups were concentrated within different categories. After thermocycling, shifts in ARI scores were observed across groups.

For ceramic brackets under both conditions, XT showed a tendency toward E in ARI scores, while GMP and SBPM exhibited a notable increase in A and a decrease to 0 in C after thermocycling.

**FIGURE 5:**
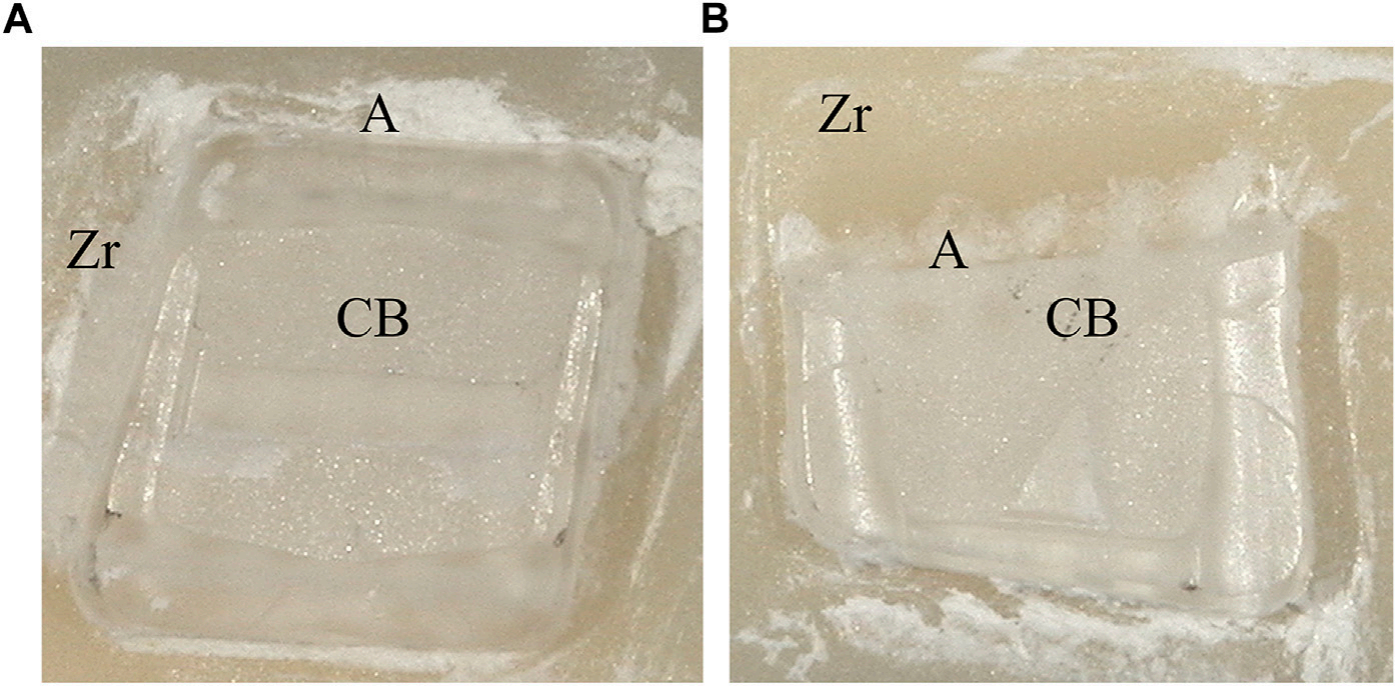
Typical bracket fractures including wings breakage **(A)** and base breakage **(B)**: Zr (zirconia), A (Adhesive), CB (ceramic bracket base).

Typical bracket fractures were observed in ceramic bracket groups after 24 h water storage, except for XT, predominantly in bracket wings with fewer base fractures in RGBU and GMP. After thermocycling, fractures were observed only in SBPM and GMP, with SBPM showing bracket base fractures. Figure 5 displays the two modes of bracket fractures.

### 3.3 Surface characterization

**FIGURE 6:**
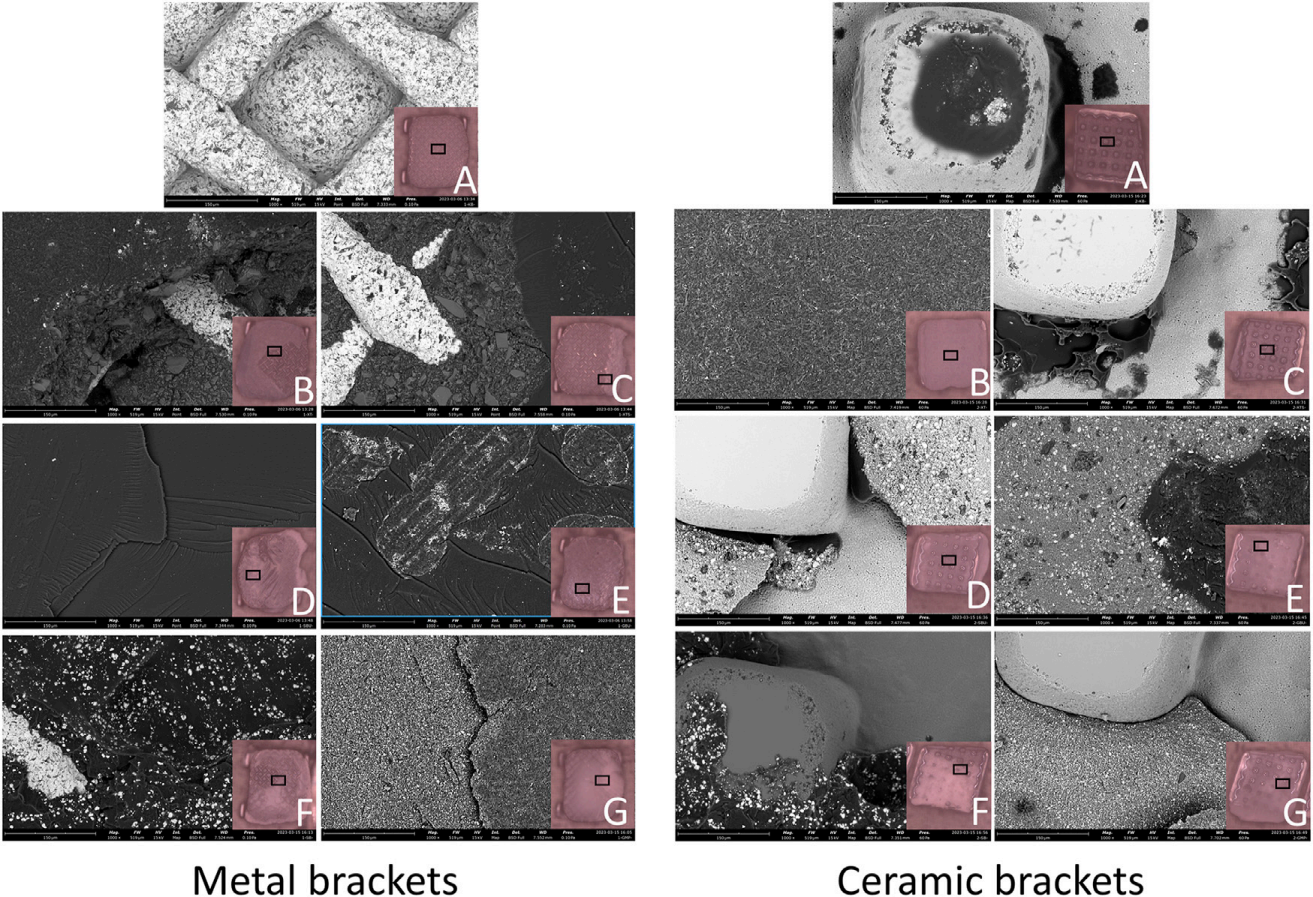
SEM photographs (1,000 × original magnification) of bracket bases: **(A)** Control Group; **(B)** XT Group; **(C)** XTS Group; **(D)** RSBU Group; **(E)** RGBU Group; **(F)** SBPM Group; **(G)** GMP Group.

**FIGURE 7:**
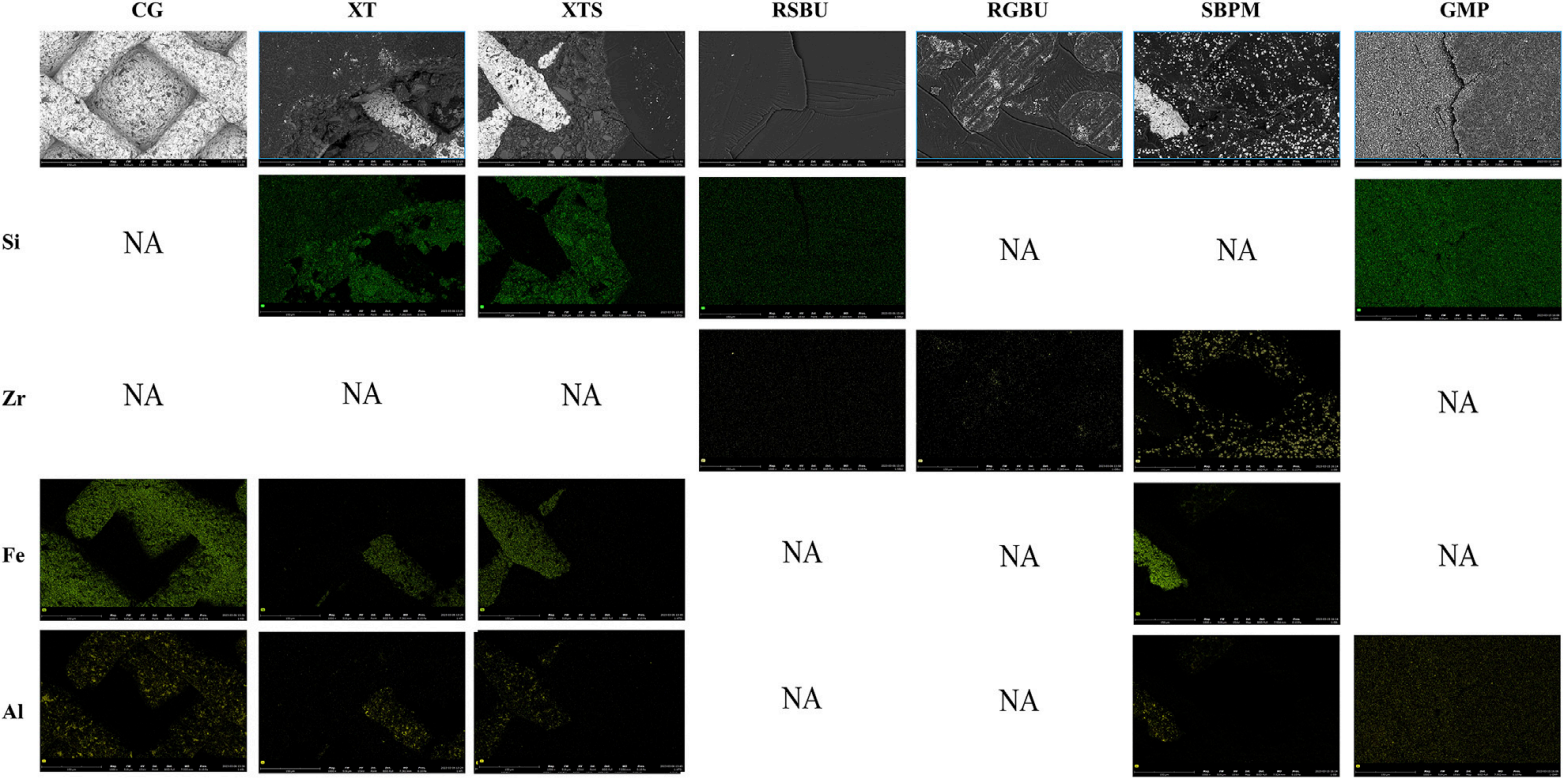
Distribution of elemental composition on the debonding base of metal brackets and ceramic brackets. CG indicates Control Group; NA indicates no applicable.

**FIGURE 8:**
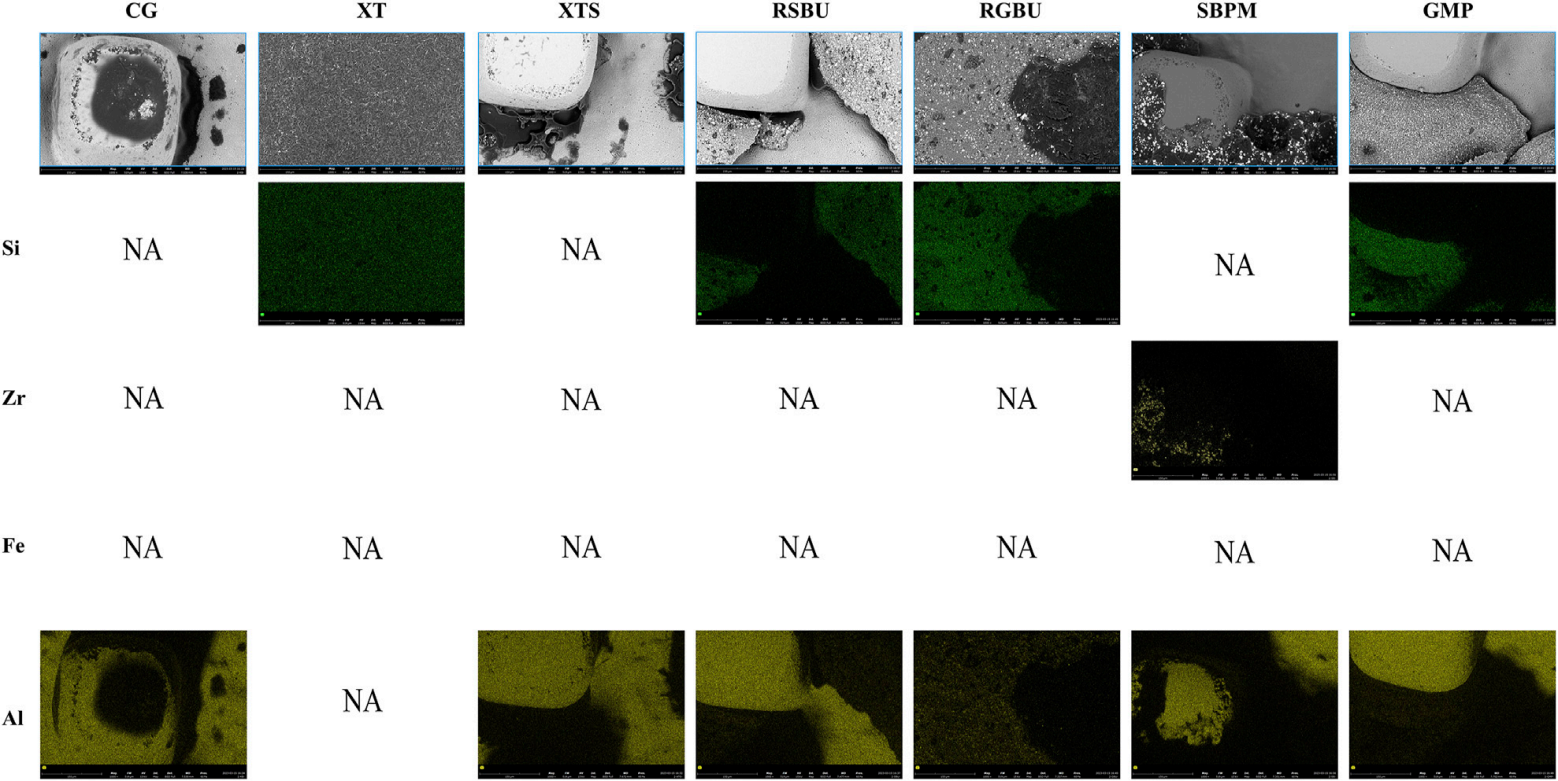
Distribution of elemental composition on the debonding base of ceramic brackets.

FE-SEM images (Figure 6) and EDS results (Figures 7, 8) indicated the presence of Si on bracket bases of XT, XTS, RSBU, RGBU, and GMP groups. Zr particles were detected in RSBU and RGBU metal bracket bases. SBPM showed Zr particles in both ceramic and metal bracket bases. Fe and Al were detected on metal bracket bases, whereas Fe was absent on ceramic bracket bases.

These observations provide insights into the elemental composition and characteristics of the bracket bases across different bonding agents and brackets used in the study.

## 4 Discussion

### 4.1 Comparison of bonding agents

The study aimed to evaluate the bond strength and durability of six bonding agents with metal or ceramic brackets on zirconia. Table 5 highlighted significant differences in shear bond strength (SBS) among bonding agents, rejecting the null hypothesis that no significant difference exists in the bond strength of different bonding agents. Typically, for optimal orthodontic efficacy, the bond strength of orthodontic brackets should ideally be within the range of 6–8 MPa at least (Uysal et al., 2004; Tecco et al., 2005). According to Table 5, except for the XT group, all metal bracket groups achieved long-term bond strengths deemed clinically acceptable. In contrast, among the ceramic bracket groups, while acceptable strength was achieved in the short term by all groups, only the SBPM group exhibited sustained durability.

Using XT as a control group for bonding brackets to enamel, it demonstrated higher SBS in ceramic brackets after 24 h of water storage compared to metal brackets. This outcome suggests the potential influence of ceramic translucence in enhancing XT resin polymerization, contributing to improved short-term bonding (Al-Hity et al., 2012; Reginato et al., 2013). However, XT’s SBS significantly decreased after thermocycling, potentially due to mismatched coefficients of shrinkage/expansion between XT resin and brackets, failing to meet the required clinical bond strength.

The XTS group, employing SBU, an universal adhesive, exhibited improved bonding performance attributed to 10-MDP’s presence, facilitating chemical bonding to zirconia via phosphate groups (Khan et al., 2017; Nagaoka et al., 2017). This bond was indicated by ARI scores concentrated on A, consistent with SBU’s ability to strengthen XTL resin-zirconia bonds.

### 4.2 Hydrophilic monomer HEMA’s effect

The absence of HEMA in GBU aimed to evaluate HEMA’s impact on bracket-zirconia bonding. RSBU (containing HEMA) for metal brackets demonstrated significantly higher SBS than HEMA-free RGBU after 24 h water storage. HEMA’s role in enhancing component miscibility and forming a uniform adhesive layer could explain RSBU’s superiority (Toledano et al., 2001; Moszner et al., 2005; Van Landuyt et al., 2005), despite its decreased SBS post-thermocycling. However, RGBU’s hydrophobic nature prevented water absorption, resulting in more stable bonding durability, aligning with earlier findings (Hu et al., 2022).

### 4.3 Impact of resin water absorption on durability

Studies have shown that HEMA-containing adhesives, due to continuous water absorption, lead to decreased bond strength post-polymerization (Ito et al., 2005; Takahashi et al., 2011). RSBU demonstrated significant SBS reduction after 5,000 cycles of thermocycling, attributed to water absorption. Conversely, RGBU’s SBS remained stable in metal brackets group, indicating its resistance to water-induced degradation. Therefore, Three-way ANOVA demonstrated a statistically significant interaction among bonding agents, storage conditions and brackets (*p* < 0.001), which was not detected in previous experiment (Hu et al., 2022).

### 4.4 Specific bonding agents’ performance

GMP, containing a self-adhesive resin cement, showed comparable SBS to SBPM in the metal bracket group after 5,000 thermocycles, suggesting GMP’s stability post-thermal stress.

SBPM exhibited significantly higher SBS for ceramic brackets, despite a notable 60% bracket breakage rate. This could be attributed to MMA resin’s water resistivity (Ikemura and Endo, 2010; Aoki et al., 2011) and the absence of silicon components in EDS analysis. Due to the absence of inorganic fillers, SBPM is typically polymerized in a linear form, resulting in better toughness compared to cross-linked polymers. On the other hand, SBPM can promote free radical polymerization of the resin using oxygen and water in the presence of TBB, a polymerization initiator, which can significantly improve the bond strength and long-term durability of ceramic brackets to zirconia porcelain (Tanaka et al., 2004; Meguro et al., 2006; Shinagawa et al., 2019). The presence of Zr in the EDS results also reveals the viewpoint. This finding is consistent with those of Shimoe et al., who also observed an increase in the [C] and [O] intensity peaks when the zirconia surface was treated with 4-META, indicating that 4-META chemically adheres to the zirconia surface effectively (Shimoe et al., 2018).

### 4.5 Influence of storage conditions

While most groups demonstrated decreased SBS after thermocycling, XTS displayed a significant SBS increase, possibly due to radical mobility enhancement in high-temperature conditions during resin solidification (Al Jabbari et al., 2014). This variance partially rejected the null hypothesis of storage conditions not affecting bonding agents’ strength.

### 4.6 Comparison of metal vs. ceramic brackets

Metal and ceramic brackets showcased comparable SBS after 24 h water storage, but ceramic brackets exhibited significantly lower SBS post-thermocycling, rejecting the null hypothesis of similar bonding durability between metal and ceramic brackets on zirconia.

### 4.7 Discussion limitations

Limitations include *in vitro* conditions not entirely mimicking oral temperatures, potentially impacting XTS’s clinical bond strength, and bracket fractures affecting measured SBS accuracy in ceramic brackets. Meanwhile, the study solely applied sandblasting treatment to zirconia surfaces, without comprehensive assessment of other factors capable of zirconia surface modification, such as laser treatment (Hou et al., 2020) and silica coating (Galvão Ribeiro et al., 2018), which have been demonstrated to have a positive impact on the bonding efficacy between zirconia and resin. The subsequent phase of research may encompass the effects of diverse surface modification treatments between orthodontic brackets and zirconia and investigate their clinical practicality.

## 5 Conclusion

1. Ceramic brackets displayed significantly lower bond strength on zirconia compared to metal brackets after thermocycling.
2. SBPM exhibited stable and sufficient bond strength between ceramic/metal brackets and zirconia under diverse storage conditions.
3. HEMA-free adhesive presented more stable bonding performance compared to HEMA-containing adhesive used in the study.

## Data Availability

The raw data supporting the conclusions of this article will be made available by the authors, without undue reservation.

## References

[B1] Alexopoulou E. Polychronis G. Konstantonis D. Sifakakis I. Zinelis S. Eliades T. (2020). A study of the mechanical properties of as-received and intraorally exposed single-crystal and polycrystalline orthodontic ceramic brackets. Eur. J. Orthod. 42, 72–77. 10.1093/ejo/cjz024 31009950

[B2] Al-Hity R. Gustin M.-P. Bridel N. Morgon L. Grosgogeat B. (2012). *In vitro* orthodontic bracket bonding to porcelain. Eur. J. Orthod. 34, 505–511. 10.1093/ejo/cjr043 21447780

[B3] Al Jabbari Y. S. Al Taweel S. M. Al Rifaiy M. Alqahtani M. Q. Koutsoukis T. Zinelis S. (2014). Effects of surface treatment and artificial aging on the shear bond strength of orthodontic brackets bonded to four different provisional restorations. Angle Orthod. 84, 649–655. 10.2319/090313-649.1 24446920 PMC8650449

[B4] Aoki K. Kitasako Y. Ichinose S. Burrow M. F. Ariyoshi M. Nikaido T. (2011). Ten-year observation of dentin bonding durability of 4-META/MMA-TBB resin cement--a SEM and TEM study. Dent. Mater J. 30, 438–447. 10.4012/dmj.2011-003 21778603

[B5] Babaee Hemmati Y. Neshandar Asli H. Falahchai M. Safary S. (2022). Effect of different surface treatments and orthodontic bracket type on shear bond strength of high-translucent zirconia: an *in vitro* study. Int. J. Dent. 2022, 1–8. 10.1155/2022/9884006 PMC9233604 35761965

[B6] Blakey R. Mah J. (2010). Effects of surface conditioning on the shear bond strength of orthodontic brackets bonded to temporary polycarbonate crowns. Am. J. Orthod. Dentofac. Orthop. 138, 72–78. 10.1016/j.ajodo.2008.08.030 20620836

[B7] Galvão Ribeiro B. R. Galvão Rabelo Caldas M. R. Almeida A. A. Fonseca R. G. Adabo G. L. (2018). Effect of surface treatments on repair with composite resin of a partially monoclinic phase transformed yttrium-stabilized tetragonal zirconia. J. Prosthet. Dent. 119, 286–291. 10.1016/j.prosdent.2017.02.014 28533011

[B8] Gautam C. Joyner J. Gautam A. Rao J. Vajtai R. (2016). Zirconia based dental ceramics: structure, mechanical properties, biocompatibility and applications. Dalton Trans. 45, 19194–19215. 10.1039/c6dt03484e 27892564

[B9] Goracci C. Di Bello G. Franchi L. Louca C. Juloski J. Juloski J. (2022). Bracket bonding to all-ceramic materials with universal adhesives. Mater. (Basel) 15, 1245. 10.3390/ma15031245 PMC8839010 35161189

[B10] Hanawa T. (2020). Zirconia versus titanium in dentistry: a review. Dent. Mater J. 39, 24–36. 10.4012/dmj.2019-172 31666488

[B11] Hellak A. Ebeling J. Schauseil M. Stein S. Roggendorf M. Korbmacher-Steiner H. (2016). Shear bond strength of three orthodontic bonding systems on enamel and restorative materials. Biomed. Res. Int. 2016, 1–10. 10.1155/2016/6307107 PMC5050387 27738633

[B12] Hou Y. Yi J. Huang Y. Gao J. Chen Y. Wang C. (2020). Effect of Er:YAG laser etching on the shear bond strength and microleakage of self-glazed zirconia ceramics. Photobiomodul Photomed. Laser Surg. 38, 289–294. 10.1089/photob.2019.4658 31944868

[B13] Hu B. Hu Y. Li X. Gao J. Sun R. Zhan D. (2022). Shear bond strength of different bonding agents to orthodontic metal bracket and zirconia. Dent. Mater J. 41, 749–756. 10.4012/dmj.2022-028 36070928

[B14] Ikemura K. Endo T. (2010). A review of our development of dental adhesives--effects of radical polymerization initiators and adhesive monomers on adhesion. Dent. Mater J. 29, 109–121. 10.4012/dmj.2009-057 20379020

[B15] Ito S. Hashimoto M. Wadgaonkar B. Svizero N. Carvalho R. M. Yiu C. (2005). Effects of resin hydrophilicity on water sorption and changes in modulus of elasticity. Biomaterials 26, 6449–6459. 10.1016/j.biomaterials.2005.04.052 15949841

[B16] Jawad Z. Bates C. Hodge T. (2015). Who needs orthodontic treatment? Who gets it? And who wants it? Br. Dent. J. 218, 99–103. 10.1038/sj.bdj.2015.51 25686425

[B17] Ju G.-Y. Lim B.-S. Moon W. Park S.-Y. Oh S. Chung S. H. (2020). Primer-treated ceramic bracket increases shear bond strength on dental zirconia surface. Mater. (Basel) 13, 4106. 10.3390/ma13184106 PMC7560292 32947875

[B18] Ju G.-Y. Oh S. Lim B.-S. Lee H.-S. Chung S. H. (2019). Effect of simplified bonding on shear bond strength between ceramic brackets and dental zirconia. Mater. (Basel) 12, 1640. 10.3390/ma12101640 PMC6566336 31137486

[B19] Khan A. A. Al Kheraif A. A. A. Jamaluddin S. Elsharawy M. Divakar D. D. (2017). Recent trends in surface treatment methods for bonding composite cement to zirconia: a reveiw. J. Adhes. Dent. 19, 7–19. 10.3290/j.jad.a37720 28195271

[B20] Lima R. B. W. Barreto S. C. Alfrisany N. M. Porto T. S. De Souza G. M. De Goes M. F. (2019). Effect of silane and MDP-based primers on physico-chemical properties of zirconia and its bond strength to resin cement. Dent. Mater 35, 1557–1567. 10.1016/j.dental.2019.07.008 31395450

[B21] Makhija S. K. Lawson N. C. Gilbert G. H. Litaker M. S. McClelland J. A. Louis D. R. (2016). Dentist material selection for single-unit crowns: findings from the national dental practice-based research network. J. Dent. 55, 40–47. 10.1016/j.jdent.2016.09.010 27693778 PMC5125852

[B22] Meguro D. Hayakawa T. Kawasaki M. Kasai K. (2006). Shear bond strength of calcium phosphate ceramic brackets to human enamel. Angle Orthod. 76, 301–305. 10.1043/0003-3219(2006)076[0301:SBSOCP]2.0.CO;2 16539558

[B23] Moszner N. Salz U. Zimmermann J. (2005). Chemical aspects of self-etching enamel-dentin adhesives: a systematic review. Dent. Mater 21, 895–910. 10.1016/j.dental.2005.05.001 16038969

[B24] Nagaoka N. Yoshihara K. Feitosa V. P. Tamada Y. Irie M. Yoshida Y. (2017). Chemical interaction mechanism of 10-MDP with zirconia. Sci. Rep. 7, 45563. 10.1038/srep45563 28358121 PMC5372092

[B25] Nistor L. Grădinaru M. Rîcă R. Mărășescu P. Stan M. Manolea H. (2019). Zirconia use in dentistry - manufacturing and properties. Curr. Health Sci. J. 45, 28–35. 10.12865/CHSJ.45.01.03 31297259 PMC6592671

[B26] Pinho M. Manso M. C. Almeida R. F. Martin C. Carvalho Ó. Henriques B. (2020). Bond strength of metallic or ceramic orthodontic brackets to enamel, acrylic, or porcelain surfaces. Mater. (Basel) 13, 5197. 10.3390/ma13225197 PMC7698487 33213042

[B27] Rauch A. Schrock A. Schierz O. Hahnel S. (2021). Material selection for tooth-supported single crowns-a survey among dentists in Germany. Clin. Oral Investig. 25, 283–293. 10.1007/s00784-020-03363-9 PMC7785551 32556660

[B28] Reginato C. F. Oliveira A. S. Kaizer M. R. Jardim P. S. Moraes R. R. (2013). Polymerization efficiency through translucent and opaque fiber posts and bonding to root dentin. J. Prosthodont Res. 57, 20–23. 10.1016/j.jpor.2012.05.003 23116926

[B29] Shimoe S. Hirata I. Otaku M. Matsumura H. Kato K. Satoda T. (2018). Formation of chemical bonds on zirconia surfaces with acidic functional monomers. J. Oral Sci. 60, 187–193. 10.2334/josnusd.17-0160 29743385

[B30] Shinagawa J. Inoue G. Nikaido T. Ikeda M. Burrow M. F. Tagami J. (2019). Early bond strengths of 4-META/MMA-TBB resin cements to CAD/CAM resin composite. Dent. Mater J. 38, 28–32. 10.4012/dmj.2017-438 30158351

[B31] Takahashi M. Nakajima M. Hosaka K. Ikeda M. Foxton R. M. Tagami J. (2011). Long-term evaluation of water sorption and ultimate tensile strength of HEMA-containing/-free one-step self-etch adhesives. J. Dent. 39, 506–512. 10.1016/j.jdent.2011.04.008 21575671

[B32] Tanaka Y. Sugaya T. Tanaka S. Kawanami M. (2004). Long-term durability of root-end sealing with 4-MEtA/MMA-TBB resin. Dent. Mater J. 23, 453–456. 10.4012/dmj.23.453 15688706

[B33] Tecco S. Traini T. Caputi S. Festa F. de Luca V. D’Attilio M. (2005). A new one-step dental flowable composite for orthodontic use: an *in vitro* bond strength study. Angle Orthod. 75, 672–677. 10.1043/0003-3219(2005)75[672:ANODFC]2.0.CO;2 16097240

[B34] Toledano M. Osorio R. de Leonardi G. Rosales-Leal J. I. Ceballos L. Cabrerizo-Vilchez M. A. (2001). Influence of self-etching primer on the resin adhesion to enamel and dentin. Am. J. Dent. 14, 205–210. 11699738

[B35] Urichianu M. Makowka S. Covell D. Warunek S. Al-Jewair T. (2022). Shear bond strength and bracket base morphology of new and rebonded orthodontic ceramic brackets. Mater. (Basel) 15, 1865. 10.3390/ma15051865 PMC8911633 35269097

[B36] Uysal T. Sari Z. Demir A. (2004). Are the flowable composites suitable for orthodontic bracket bonding? Angle Orthod. 74, 697–702. 10.1043/0003-3219(2004)074<0697:ATFCSF>2.0.CO;2 15529507

[B37] Van Landuyt K. L. De Munck J. Snauwaert J. Coutinho E. Poitevin A. Yoshida Y. (2005). Monomer-solvent phase separation in one-step self-etch adhesives. J. Dent. Res. 84, 183–188. 10.1177/154405910508400214 15668338

[B38] Vult von Steyern P. Bruzell E. Vos L. Andersen F. S. Ruud A. (2022). Sintering temperature accuracy and its effect on translucent yttria-stabilized zirconia: flexural strength, crystal structure, tetragonality and light transmission. Dent. Mater 38, 1099–1107. 10.1016/j.dental.2022.04.023 35570007

[B39] Zarone F. Di Mauro M. I. Ausiello P. Ruggiero G. Sorrentino R. (2019). Current status on lithium disilicate and zirconia: a narrative review. BMC Oral Health 19, 134. 10.1186/s12903-019-0838-x 31272441 PMC6610968

[B40] Zhang Y. Lawn B. R. (2018). Novel zirconia materials in dentistry. J. Dent. Res. 97, 140–147. 10.1177/0022034517737483 29035694 PMC5784474

