# Corrigendum: The possibility of clinical bonding between metal/ceramic brackets to zirconia: *in vitro* study

**DOI:** 10.3389/fbioe.2024.1423030

**Published:** 2024-05-15

**Authors:** Yichun Hu, Jiayang Gao, Xinyue Huang, Yutong Li, Ziyi Chen, Desong Zhan, Hidehiko Sano, Ricardo M. Carvalho, Jiale Fu

**Affiliations:** ^1^ School and Hospital of Stomatology, China Medical University, Shenyang, China; ^2^ Department of Dental Materials Science, The Second Department of Prosthodontics, School and Hospital of Stomatology, China Medical University, Shenyang, China; ^3^ Department of Restorative Dentistry, Division of Oral Health Science, Faculty of Dental Medicine, Hokkaido University, Sapporo, Japan; ^4^ Department of Oral Biological and Medical Sciences, Division of Biomaterials, Faculty of Dentistry, University of British Columbia, Vancouver, BC, Canada

**Keywords:** shear bond strength (SBS), ceramic bracket, zirconia, resin cement, metal bracket, storage condition

In the published article, there was an error. A sentence was included which inaccurately describes **Figures 3 and 4**.

A correction has been made to **3.2 Failure modes**, Paragraph 2. This sentence previously stated:

“For ceramic brackets under both conditions, XT showed a tendency toward E in ARI scores, while SBPM shifted toward C after thermocycling. Other groups displayed varied tendencies across scores.”

The corrected sentence appears below:

“For ceramic brackets under both conditions, XT showed a tendency toward E in ARI scores, while GMP and SBPM exhibited a notable increase in A and a decrease to 0 in C after thermocycling.”

The authors apologize for this error and state that this does not change the scientific conclusions of the article in any way. The original article has been updated.

